# Does the surgeon still have a role to play in the diagnosis and management of lymphomas?

**DOI:** 10.1186/1477-7819-6-13

**Published:** 2008-02-04

**Authors:** Gareth Morris-Stiff, Peipei Cheang, Steve Key, Anju Verghese, Timothy J Havard

**Affiliations:** 1 Department of Surgery, Royal Glamorgan Hospital, Ynysmaerdy, Llantrisant, UK; 2 Department of Pathology, Royal Glamorgan Hospital, Ynysmaerdy, Llantrisant, UK

## Abstract

**Background:**

Over the course of the past 40 years, there have been a significant number of changes in the way in which lymphomatous disease is diagnosed and managed. With the advent of computed tomography, there is little role for staging laparotomy and the surgeon's role may now more diagnostic than therapeutic.

**Aims:**

To review all cases of lymphoma diagnosed at a single institution in order determine the current role of the surgeon in the diagnosis and management of lymphoma.

**Patients and methods:**

Computerized pathology records were reviewed for a five-year period 1996 to 2000 to determine all cases of lymph node biopsy (incisional or excisional) in which tissue was obtained as part of a planned procedure. Cases of incidental lymphadenopathy were thus excluded.

**Results:**

A total of 297 biopsies were performed of which 62 (21%) yielded lymphomas. There were 22 females and 40 males with a median age of 58 years (range: 19–84 years). The lymphomas were classified as 80% non-Hodgkin's lymphoma, 18% Hodgkin's lymphoma and 2% post-transplant lymphoproliferative disorder. Diagnosis was established by general surgeons (n = 48), ENT surgeons (n = 9), radiologists (n = 4) and ophthalmic surgeons (n = 1). The distribution of excised lymph nodes was: cervical (n = 23), inguinal (n = 15), axillary (n = 11), intra-abdominal (n = 6), submandibular (n = 2), supraclavicular (n = 2), periorbital (n = 1), parotid (n = 1) and mediastinal (n = 1). Fine needle aspiration cytology had been performed prior to biopsy in only 32 (52%) cases and had suggested: lymphoma (n = 10), reactive changes (n = 13), normal (n = 5), inadequate (n = 4). The majority (78%) of cervical lymph nodes were subjected to FNAC prior to biopsy whilst this was performed in only 36% of non-cervical lymphadenopathy.

**Conclusion:**

The study has shown that lymphoma is a relatively common cause of surgical lymphadenopathy. Given the limitations of FNAC, all suspicious lymph nodes should be biopsied following FNAC even if the FNAC is reported normal or demonstrating reactive changes only. With the more widespread application of molecular techniques, and the development of improved minimally-invasive procedures, percutaneous and endoscopic techniques may come to dominate, however, at present; the surgeon still has an important role to play in the diagnosis if not treatment of lymphomas.

## Background

**Figure 1:**
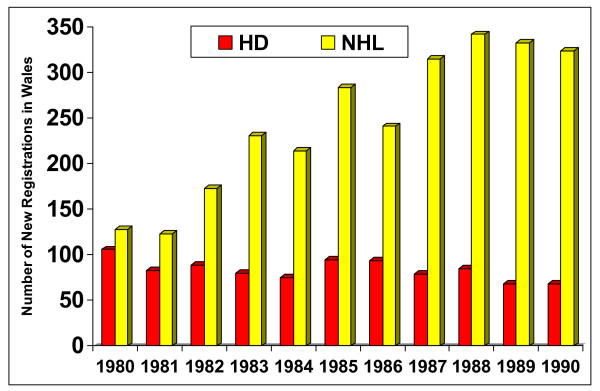
Diagnosis of lymphoma in Wales over the period 1980–1990. HD = Hodgkin's disease, NHL = Non-Hodgkin's lymphoma.

Lymphomas are a heterogeneous family of malignant neoplasia of the reticuloendothelial system, which may be divided into two main subtypes; Hodgkin's lymphoma (HL), eponymous to the nineteenth century Guy's pathologist Thomas Hodgkin, and non-Hodgkin's lymphoma (NHL). The incidence of NHL increased over the 1980s decade from 120 to 320 registrations per year whereas the incidence of HL has remained static at around 80 cases per year in Wales as illustrated in Figure 1[1].

The surgeon's role in the diagnosis and management of lymphomas, in particular HL, was stimulated by a report from Stanford University in the late 1960s which showed that the performance of a staging laparotomy altered the stage of disease in 42% of cases, up regulating in 28% and down regulating in 14% of cases [2]. The procedure consisted of liver and lymph node biopsies together with splenectomy. In addition to allowing accurate staging, the splenectomy was believed to debulk the disease mass and offer a more precise target for radiotherapy.

The advent of computed tomography brought about the demise of staging laparotomies and splenectomy is now limited to symptomatic splenomegaly and occasionally hyposplenism. Computed tomography is rapid, non-invasive and allows assessment of both thoracic and abdominal compartments. However, a tissue diagnosis is still required to allow accurate cellular classification of the lymphomas.

Fine needle aspiration cytology (FNAC) was developed at the turn of the century and has become a popular diagnostic tool as it is rapid, painless, safe, inexpensive, does not require any anaesthetic or hospital admission and leaves no scar [3]. In addition to confirming the diagnosis of lymphomas, one of the important roles of FNAC is the exclusion of metastatic squamous carcinoma as this requires an alternative therapeutic approach. There is a question as to the accuracy of FNAC in the diagnosis of lymphomas as the tumours often contain malignant and reactive elements and the FNAC may only have sampled the reactive regions leading to false negative results. Another disadvantage of FNAC of lymphomas is that it does not provide the cellular architecture required for the accurate subtyping of the lymphoma.

As a result of the deficiencies of FNAC, lymph node excision is required and is the recommended second line diagnostic procedure. In addition to providing a greater volume of tissue for histological evaluation subtype classification, it also provides a baseline against which the effects of chemotherapy may be judged.

The aim of this study was to examine whether the 21^st ^century surgeon still has a role to play in the diagnosis and management of lymphoma.

## Patients and methods

The study was a retrospective study of all patients undergoing lymph node biopsy at the Royal Glamorgan Hospital (formerly known as East Glamorgan Hospital) for the five-year period 1996 to 2000. Patients were identified from the computerised records of the pathology department. All cases of lymph node biopsy were collected (excisional and incisional) however patients in whom lymphadenopathy was an incidental finding were excluded and thus the cohort consisted of patients in whom the aim of surgery was lymph node biopsy.

For each patient the following information was collected: patient demographics, location of lymphadenopathy, findings of lymph node biopsy, performance or not of FNAC and findings of FNAC.

## Results

**Figure 2:**
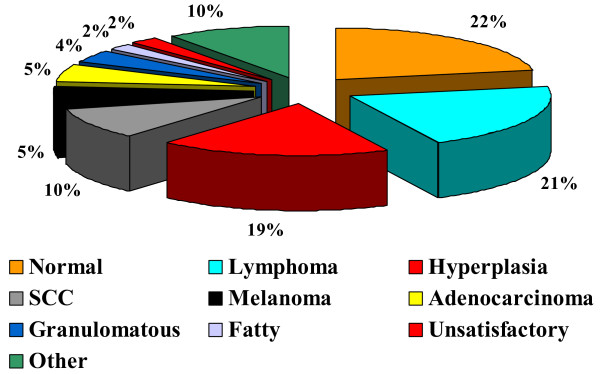
Findings of lymph node biopsies (n = 279).

The study population comprised 297 patients undergoing lymph node biopsy (Figure 2). Lymphoma was confirmed in 62 patients, representing 21% of all biopsies. There were 40 males and 22 females of median age 58 years (range 19–84 years). The lymphomas were classified into 80% NHL, 18% HL and 2% post-transplant lymphoproliferative disorder.

Diagnosis was established mainly by general surgeons (n = 48), ENT surgeons (n = 9), radiologists (n = 4) and ophthalmic surgeons (n = 1). The anatomical distribution of the excised lymph nodes is detailed in Table 1. The commonest locations for lymphadenopathy were cervical (n = 23), inguinal (n = 15), and axillary (n = 11).

**Table 1:** Anatomical location of lymphomatous lymph nodes (n = 62)

**Anatomical location**	**Number of cases**
Cervical	23
Inguinal	15
Axillary	11
Intra-abdominal	6
Supraclavicular	2
Submandibular	2
Parotid	1
Peri-orbital	1
Mediastinal	1

Fine needle aspiration cytology had been performed prior to biopsy in only 32 (52%) cases out of the total of 62 with a final diagnosis of lymphoma. The findings of FNAC were: lymphoma (n = 10); reactive changes (n = 13); normal (n = 5); inadequate (n = 4). The remaining 30 patients proceeded to biopsy without FNAC. FNAC was performed in 18 of 23 patients with cervical lymphadenopathy but in only 14 of 39 of individuals with non-cervical lymphadenopathy. The time interval between performance of FNAC and histological confirmation of the biopsy specimens was less than one month in 81% of cases and less than six weeks in all cases. In cases of delay more than one month, delays were due to patient non-compliance.

## Discussion

The study has confirmed that lymphoma is a common cause of surgical lymphadenopathy, representing the histological diagnosis in 21% of all lymph node biopsy specimens. The ratio of HL to NHL in this study was identical to the current trend in lymphoma incidence in Wales with a ratio of 1:4 [1].

The locations of lymphomatous nodes corresponded to the distribution of lymphadenopathy as a whole, with the majority of palpable nodes being in the cervical, inguinal and axillary chains and as such were amenable to simple excision. The majority of lymph node biopsies were performed mainly by general surgeons whilst ENT and ophthalmic surgeons performed a total of ten biopsies. The remaining four lymphomas were biopsied using ultrasound-guidance by radiologists.

Fine needle aspiration cytology was performed in little over half of the cases although this was performed in 81% of head and neck lymphadenopathy in accordance with practice guidelines [4]. The importance of performing an FNAC in patients with cervical lymphadenopathy prior to embarking on an excisional biopsy relates to the fact that, for those patients found to have squamous carcinoma metastases from a head and neck primary, open biopsy leads to a significantly higher local treatment failure rate which may in turn be associated with an adverse effect on survival [5,6].

The accuracy of FNAC in the diagnosis of lymphoma has previously been questioned [7]. The lymphomatous process may involve the node focally and may not involve all the nodes that appear to be enlarged. Other factors that influence the diagnostic specificity and sensitivity of FNAC in the diagnosis of lymphoma include; necrosis in involved nodes; the presence of dual pathology and sclerosis/fibrosis in involved nodes leading to insufficient diagnostic material.

Other disadvantages of FNAC are lack of material for an accurate typing of lymphoma due to lack of tissue for immunohistochemistry [5]. Low grade lymphomas are difficult to diagnose even on excisional biopsies and special staining techniques are required to differentiate between a florid follicular hyperplasia and a follicular lymphoma.

In this study, lymphomas were correctly identified by FNAC in only 31% of cases. The commonest diagnosis, in 40% of FNACs was reactive changes whilst the remaining cases were equally divided between normal and inadequate. All patients with FNACs not diagnostic of lymphoma went on to lymph node biopsy because of suspicious clinical histories or persisting lymphadenopathy. The performance of FNAC was not regarded as being compulsory at the start of this observational study but became standard practice, and more recently the performance of FNAC under ultrasound-guidance was introduced in order to maximize the likelihood of correctly targeting the suspicious lymph node.

The uses of flow cytometry, immunohistochemistry, and molecular studies such as polymerase chain reaction and fluorescent in-situ hybridization have significantly increased the yield of FNAC [8-10]. Furthermore, the more recent introduced technique of core biopsy has been shown to be of benefit over FNAC in the diagnosis of lymphoma especially when performed under ultrasound-guidance combined with advanced molecular techniques [11-13].

One area not explored by this study but which may be of increasing importance in the future is the role of endoscopy and laparoscopy in obtaining biopsy material. The advent of endoscopic ultrasound-guided FNAC allows targeting of mediastinal and intra-abdominal lymphadenopathy, which can be performed without the morbidity associated with trans-cavity radiological sampling or open surgical biopsy [14-16]. For lesions outside the reach of the endoscope, laparoscopy may play an increasing role [17,18] as it allows access to perihepatic and perisplenic in addition to retroperioneal lymphadenopathy. Thus upper gastrointestinal surgeons with training in these techniques may have an increasing role in the diagnosis of lymphomas. In cases of intrathoracic lympahadopathy, newer minimally-invasive techniques such as mediasinoscopy; thoracoscopy are also now well established and provide adequate tissue for sub-typing [19]. Although not performed by 'general surgeons', they do represent a surgical biopsy.

## Conclusion

All patients presenting with lymphadenopathy should undergo FNAC, this being of critical importance for cervical lesions as lymphadenopathy presenting in this region may represent metastases from primary squamous cell carcinomas of the head and neck. Given the limitations of FNAC, all suspicious lymph nodes should be biopsied if the FNAC is reported normal or demonstrates reactive changes only, this being performed mainly by general surgeons. Thus at present the 'surgeon' still has a role to play in the diagnosis of lymphoma.

Advancements in diagnostic methods has meant that many superficial lesions traditionally requiring open excision biopsy may now be able to be diagnosed accurately by image-guided core biopsy, thus reducing the role of the surgeon. However, on the contrary, deep-seated lesions previously targeted by radiologists may now be more accurately approached by minimally-invasive surgical techniques and so a new role is likely to evolve for the surgeon in the diagnosis of lymphoma.

## Competing interests

The author(s) declare that they have no competing interests.

## Authors' contributions

**GMS **developed the concept, and prepared the draft manuscript. **PC **and **SK **provided the pathological data and helped in preparing the manuscript, **AV **and **TGH **reviewed and edited the manuscript and helped in preparing the final version. All authors read and approved final manuscript.
